# Clinical Features, Radiological Characteristics, and Outcomes of Patients With Intracranial Alveolar Echinococcosis: A Case Series From Tibetan Areas of Sichuan Province, China

**DOI:** 10.3389/fneur.2020.537565

**Published:** 2021-01-15

**Authors:** Sisi Li, Jiani Chen, Yongqiao He, Yongyi Deng, Jie Chen, Wenyu Fang, Zhamu Zeren, Yadong Liu, Ammar Taha Abdullah Abdulaziz, Bo Yan, Dong Zhou

**Affiliations:** ^1^Department of Neurology, West China Hospital, Sichuan University, Chengdu, China; ^2^Department of Neurology, Ganzi Tibetan Autonomous Prefecture People's Hospital, Kangding, China

**Keywords:** intracranial alveolar echinococcosis, *Echinococcus multilocularis*, mortality, follow-up, outcomes

## Abstract

**Objectives:** Intracranial alveolar echinococcosis (IAE), a zoonotic disease, is a critical health problem in the Tibetan region. We aimed to describe the clinical and radiological characteristics and outcomes among patients with IAE.

**Methods:** We screened patients diagnosed with IAE between March 2015 and May 2019 at the Ganzi Tibetan Autonomous Prefecture People's Hospital. Detailed demographics, clinical characteristics, neuroimaging features, and outcomes were recorded.

**Results:** A total of 21 patients with an average age of 44.1 ± 12.7 years were included. Thirteen (61.9%) patients were male. The most common chief neurological complaint was headache (*n* = 17, 81.0%), followed by dizziness, seizure, visual disturbances, hemiparesis, disturbed consciousness, and dysphasia. All the patients had coexisting liver localizations. The typical neuroimaging features of IAE on cerebral magnetic resonance imaging scans showed obvious low-signal shadow with multiple small vesicles inside the lesions on T2-weighted images and FLAIR images. The pathological HE staining demonstrates vesicular lesions with several internal sacs. For hepatic alveolar echinococcosis (AE), the hepatic portal was invaded in six (28.6%) patients, and the portal vein (*n* = 5, 23.8%) was the mostly commonly involved vessel. As for treatment, 11 patients (52.4%) had poor compliance with albendazole. The duration of patients taken albendazole ranged from 2 months to 3 years. Cerebral AE surgery was performed in 11 patients, five of them underwent partial resection of AE lesions, and six patients received total resection. One patient with primary IAE underwent radical surgery. Ten patients (47.6%) died during the follow-up for a mean of 21.7 ± 11.9 (3–46) months. In total, 28.9% of the patients died within 5 years, and 71.6% died within 10 years. The median interval between the date of diagnosis as AE and death was 84 (19–144) months.

**Conclusion:** Despite substantial advances in diagnostic and therapeutic methods, the treatment of IAE remains difficult and results in unsatisfactory outcomes. The major critical issue is surgical treatment of IAE although the disease is disseminated. Besides, lifelong albendazole would be indicated, but most patients had poor medication compliance. It is important to educate patients about the necessity of medical treatment.

## Introduction

Echinococcus, which causes severe echinococcosis or hydatid disease, is a zoonotic tapeworm found in several parts of the world (1). Cystic echinococcosis (CE) and alveolar echinococcosis (AE) are caused by *Echinococcus granulosus* and *Echinococcus multilocularis*, respectively (2). The two cestodes differ in their life cycles, morphology, and epidemiology. *E. granulosus* is more adapted to high-temperature weather, whereas *E. multilocularis* is more adapted to colder climates (3). The distribution of AE is confined to the Northern Hemisphere, principally in areas of North America, west-central Europe, the Near East, Siberia, Central Asia, Japan, and China (4–6). AE has a relatively low incidence considered fatal (1, 7). Risk factors associated with AE infections include dogs, fox-skin products or fox hunting, and sources of drinking water (8, 9). The role of wild rodents and dogs in the transmission of *E. multilocularis* to humans appears to be significant (10). The diagnosis of AE is complicated by prolonged incubation time, various clinical manifestations, and mimicking of differential diagnosis (4). The primary organ affected is the liver, AE is a pathogenic “tumor-like” disease with the potential for local and distant metastases, usually to the lungs or even the brain (11). The involvement of the brain is generally perceived as the terminal phase of the disease (12). Intracranial alveolar echinococcosis (IAE) is rare, accounting for only 1% of patients with hepatic AE (7). Studies on IAE are rare and usually published as case reports (11, 13–15). The clinical features and outcomes of the condition are not well-understood in the absence of a systematic study. In this retrospective study with follow-up, we aimed to gain further understanding of IAE regarding clinical features, neuroimaging, and outcomes.

## Materials and Methods

### Study Design

This is a retrospective case series study using the electronic medical records of all patients diagnosed with IAE in the Ganzi Tibetan Autonomous Prefecture People's Hospital between March 2015 and May 2019. This hospital is a tertiary hospital located in Kangding City, the capital of Ganzi Tibetan Autonomous Prefecture, Sichuan province, China. In total, 106 patients were clinically diagnosed as IAE, of which 85 were diagnosed as probable or possible cases, and only 21 patients were diagnosed as confirmed cases based on the recommendations of the World Health Organization's Informal Working Group on Echinococcosis (WHO-IWGE) (16). All the 21 patients were diagnosed using magnetic resonance imaging (MRI)/computed tomography (CT) scans, and diagnoses were confirmed by surgery. Serological tests were used to detect the human echinococcus antibody IgG [Enzyme-linked Immunosorbent Assay (ELISA)].

### Ethics Statement

The study's objectives and procedures were approved by the Ethics Committee of Ganzi Tibetan Autonomous Prefecture People's Hospital. All patients participating in the study or their guardians provided written informed consent.

### Data Collection

Data were collected by reviewing the medical records. Demographic information included sex, age, ethnic group, marital status, county of residence, education level, and employment information. Clinical data included symptoms, patient age at disease onset, the duration of AE, the outcome of IgG echinococcus antibody tests, neuroimaging features, AE surgery information, medical treatment, and outcomes. Illiterate is used to refer to a person who had never been to school. Follow-up of the survivors was conducted by telephone in September 2017, September 2018, and September 2019. Overall, 21 patients participated in the follow-up for a mean of 21.7 ± 11.9 (3–46) months.

### Statistical Analysis

IBM SPSS software version 22.0 (IBM Corp., Armonk, NY, USA) was used for the statistical analyses. Descriptive analyses were used to examine the sociodemographic and clinical data; categorical variables are shown as percentages (%), and continuous variables are presented as the mean (± deviation) or median (interquartile range). MRIs were performed using a 1.5 T imaging system (Philips Achieva, Holland & Sonata, Siemens Healthcare, Erlangen, Germany).

## Results

### Demographic and Clinical Information

One hundred and six patients clinically diagnosed with IAE were collected from March 2015 to May 2019, of which 99 were followed-up with a mean duration of 23.3 ± 13.74 (2–54) months. After exclusion of 85 possible cases of IAE without pathological information, a total of 21 patients diagnosed with IAE were enrolled in this study and received follow-up for a mean duration of 21.7 ± 11.9 (3–46) months. Detail information about age, sex and main symptoms of all the 21 patients were showed in Tables 1–3. The average age was 44.1 ± 12.7 years; the mean age at AE onset was 41.8 ± 13.3 years. Thirteen (61.9%) patients were male and eight (38.1%) patients were female. Among the 21 patients with IAE, 1(4.8%) was Han, and 20 (95.2%) were Tibetan. All the 21 patients lived in Tibet, Sichuan province. Seven patients came from Dege county, five came from Shiqu county, four came from Sertar county, two came from Ganzi county, and the remaining three came from Kangding county, Litang county, and Xinlong county. Twenty (95.2%) patients were illiterate, and one (4.8%) had a primary-school education. Eleven (52.4%) patients were farmers, four (19.0%) were herdsmen, five (23.8%) were monks or Buddhist nuns, and one (4.8%) was retired.

**Table 1:** Clinical data of patients who underwent cerebral alveolar echinococcosis (AE) operation.

**Case no**.	**Age/ sex**	**Main symptoms**	**Cerebral location**	**Other location**	**Treatment**	**Cerebral AE surgery**	**Degree of cerebral surgery**	**Duration of ABZ therapy (Regularity)**	**AE duration (mths)^a^**	**Max mRS/ GCS at post-IOp**	**Time to recurrence**	**Outcomes (follow-up^b^)**
1	23/M	Visual disturbances	Multiple	Liver, Kidney	IOp + ABZ	Right cerebellar lobectomy	Partially removed	1 year (Irregular)	39 mths	1/15	NA	Alive (26 mths) Decreased vision
3	30/M	Headache, Diplopia, Vomiting	Right PO.	Liver, Lung	ABZ + IOp	Right occipital and parietal lobectomy	Completely removed	2 years (Irregular)	36 mths	2/15	None	Alive (10 mths)
11	54/F	Headache, Decreased vision	Bilateral O.	Liver, Lung	ABZ + IOp	Bilateral occipital lobectomy	Completely removed	2 years (Irregular)	36 mths	3/15	None	Alive (5 mths) Decreased vision
12	60/M	Hemiparesis, Dysphasia	Multiple	Liver	IOp + ABZ	Left frontal, occipital and parietal lobectomy	Partially removed	3 months (Regular)	3 mths	4/9	NA	Alive, poor condition (3 mths) Epilepsy
14	31/M	Headache, Vomiting	Multiple	Liver, Lung	IOp	Left cerebellar lobectomy	Partially removed	None	30 mths	NA	NA	Dead (30 mths)
16^c^	51/M	Headache	Right T.	Liver	IOp	Right occipital and temporal lobectomy	Completely removed (radical)	Unclear (Irregular)	144 mths	NA	11 years	Dead (22 mths)

*F, female; M, male; T, temporal lobe; O, occipital lobe; PO, parieto-occipital lobe; AE, alveolar echinococcosis; IOp, intracranial AE operation; HOp, hepatic AE operation; ABZ, albendazole; Max, maximum; mRS, modifified Rankin Scale; GCS, Glasgow coma scale; mth, month; NA, not applicable*.

a *Course of disease, duration from AE onset until death or last follow-up (months)*.

b *Follow-up, duration of follow-up after discharge (months)*.

c *The patient was diagnosed with primary IAE and underwent cerebral AE surgery. Recurrence of brain and liver was found 11 years after the operation*.

Headache (*n* = 17, 81.0%) was the most common chief neurological complaint, followed by dizziness (*n* = 11, 52.4%), seizure (*n* = 4, 19.0%), visual disturbances (*n* = 3, 14.3%), hemiparesis (*n* = 2, 9.5%), disturbed consciousness (*n* = 2, 9.5%), and dysphasia (*n* = 1, 4.8%). Non-neurological chief complaints included nausea (*n* = 10, 47.6%), vomiting (*n* = 8, 38.1%), abdominal discomfort (*n* = 7, 33.3%), feeble (*n* = 3, 14.3%), and cough (*n* = 1, 4.8%). Serological tests were performed in 16 patients and showed that 14 were positive and two were weekly positive. As for complications in this cohort, pneumonia (*n* = 9, 42.9%) was the most common comorbidity. Less common complications included hypoalbuminemia in four patients, electrolyte disturbance in three patients, and abnormal hepatic function in three patients. In addition, there were comorbidities of tuberculosis in three patients, chronic hepatitis B in two patients, and syphilis in one patient.

### Imaging Features and Pathological Finding

**Figure 1:**
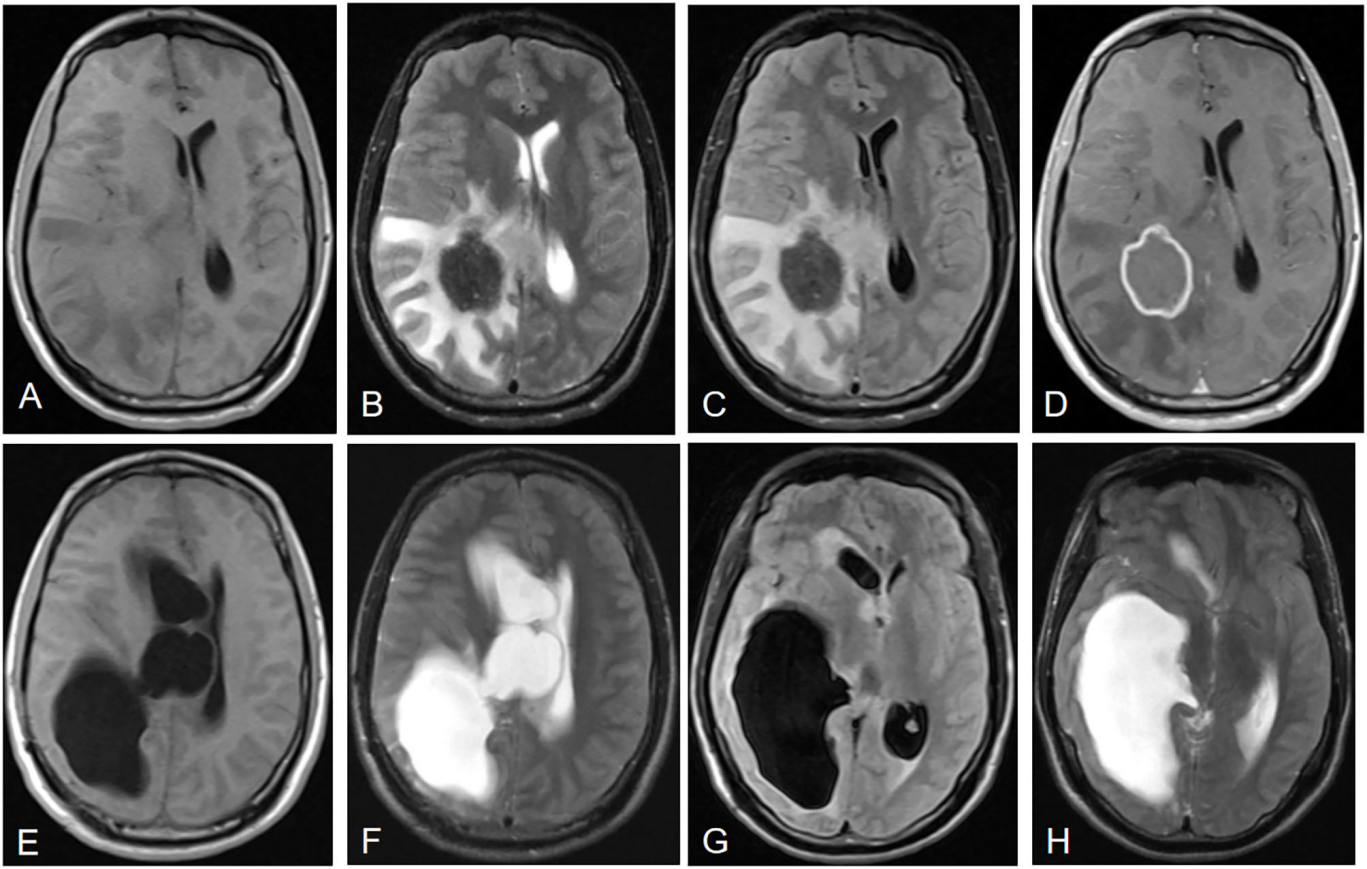
Neuroimaging features of IAE. Case 3: **(A)** T1-weighted imaging shows a slightly higher signal mass. **(B)** T2-weighted imaging reveals a heterogeneous low signal mass with peripheral high signal intensity, suggesting edema. **(C)** Fluid-attenuated inversion recovery imaging (FLAIR) reveals a heterogeneous low signal mass. **(D)** Postcontrast axial T1-weighted imaging shows cauliflower peripheral gadolinium-contrast enhancement at the lesions. **(E–H)** Right lateral ventricular dilatation and hydrocephalus occurred 7 months after right parietal and occipital lobe resection.

**Figure 2:**
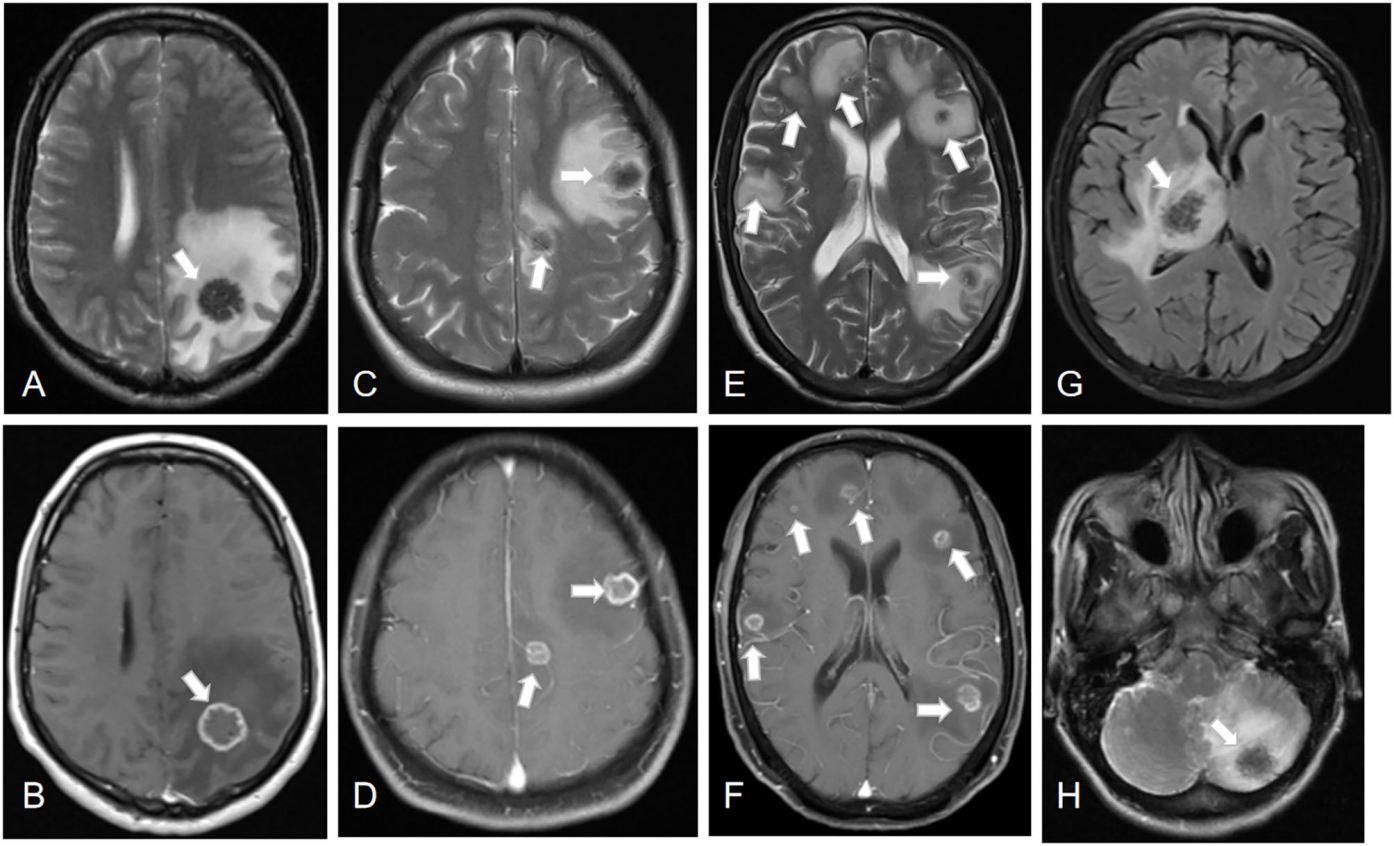
Lesions of IAE at different sites (white arrows). Case 8: **(A,B)** A cerebral lesion in the left parietal lobe infiltrating the surrounding tissue. Case 13: **(C,D)** Two cerebral lesions in the left parietal lobe and frontal lobe. Case 4: **(E,F)** Multiple cerebral lesions of IAE. Case 21: **(G)** A cerebral lesion in the right thalamus. Case 17: **(H)** A cerebral lesion in the left cerebellum.

All the 21 patients with IAE had coexisting liver localizations; 10 patients had multiple organ involvement with liver and lungs (*n* = 8, 38.1%), liver and kidney (*n* = 1, 4.8%), or liver, lung, and kidney (*n* = 1, 4.8%). The cerebral MRI showed multiple cerebral localizations in 12 (57.1%) patients. Most of the lesions occurred in the cerebral hemisphere: 15 (71.5%) cases were found in the parietal lobe areas, 10 (47.6%) in the temporal lobe, 14 (66.7%) in the occipital lobe, and seven (33.3%) in the frontal lobe. In addition, six (28.6%) cases were in the cerebellum, three (14.3%) were in the basal ganglia, and one (4.8%) was in the brain stem. Cerebral cranial MRI performance is showed in Figures 1, 2. T1-weighted imaging revealed a slightly higher signal mass. T2-weighted imaging and fluid-attenuated inversion recovery imaging (FLAIR) revealed obvious low-signal shadow with multiple small vesicles inside the lesions. Peripheral high signal intensity suggestive of edema was also shown on T2-weighted images and FLAIR images. Postcontrast axial T1-weighted images showed cauliflower peripheral gadolinium-contrast enhancement at the lesions.

**Figure 3:**
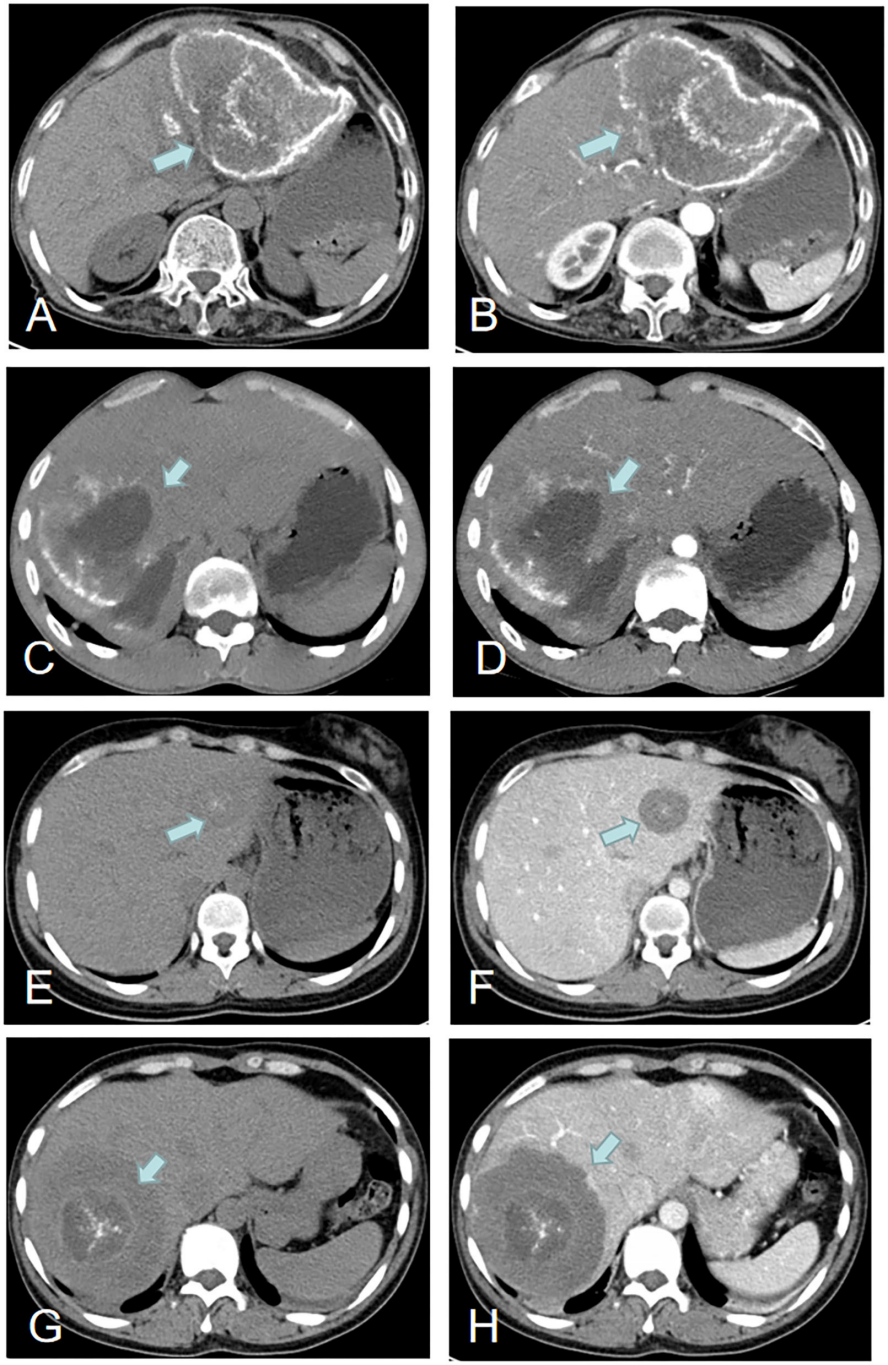
Imaging features of hepatic AE (green arrows). Case 22: **(A,B)** A hepatic lesion in the left lobe of the liver with peripheral calcification. Case 3: **(C,D)** A hepatic lesion in the right lobe of the liver with partial peripheral calcification and necrosis and liquefaction in the center of the hepatic lesion. Case 18: **(E,F)** A hepatic lesion in the left lobe with central areas of calcification. Case 13: **(G,H)** A hepatic lesion in the right lobe with central areas of calcification.

Among the 21 patients with hepatic AE, the hepatic lesions in 16 (76.2%) were solitary, and the hepatic portal was invaded in six (28.6%) patients. The portal vein (*n* = 5, 23.8%) was the most commonly involved vessel, the hepatic vein was invaded in four (19.0%) patients, and the inferior vena cava was invaded in three (14.3%) patients. Abdominal computed tomography (CT) performance for hepatic AE is showed in Figure 3. Calcification is usually found in the peripheral or central areas of hepatic AE lesions. However, there was no enhancement of hepatic AE lesions after intravenous injection of contrast media.

**Figure 4:**
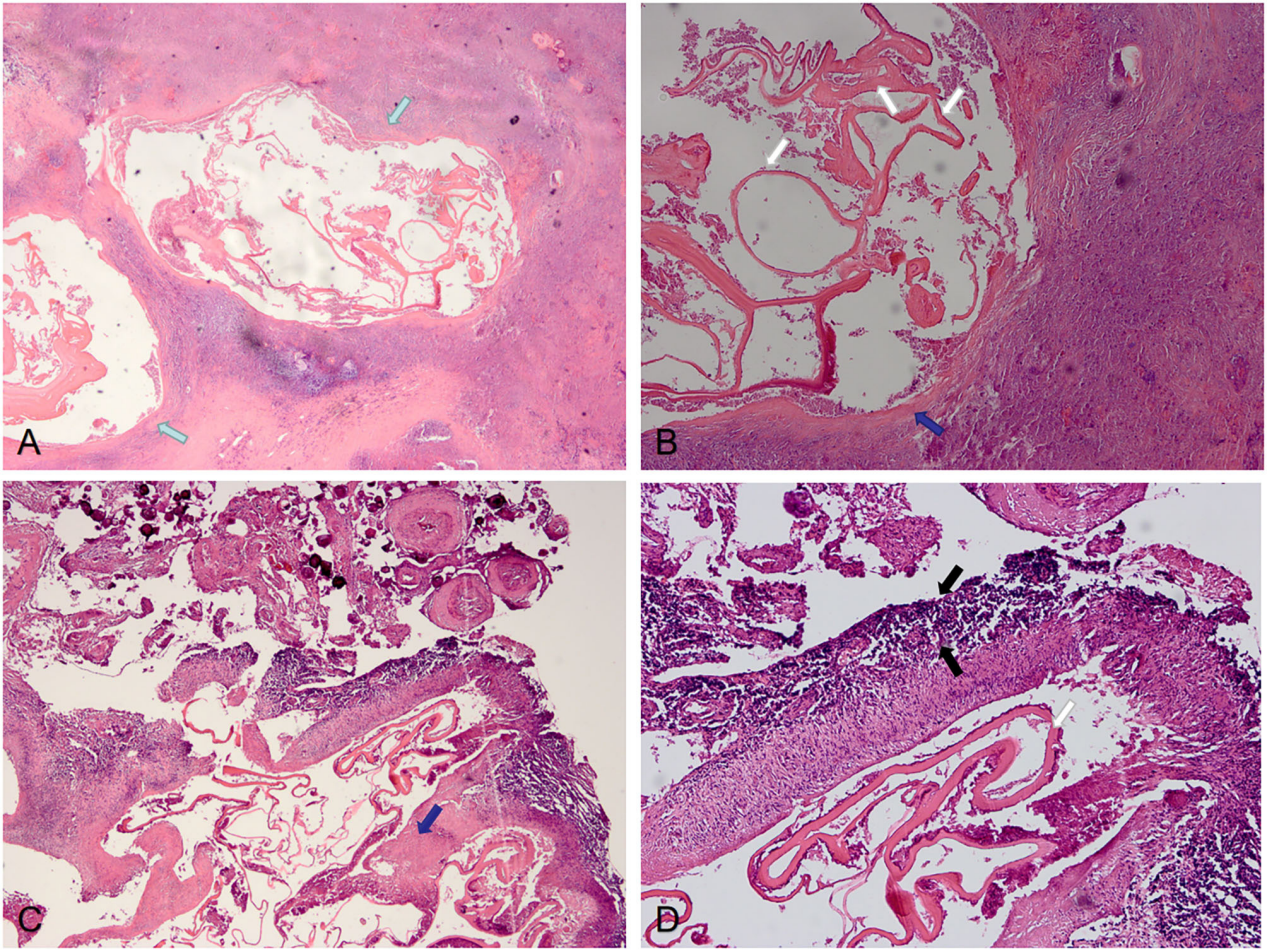
Pathological features of IAE. Case 21: **(A)** Two lesions are visible in the parenchyma (green arrows), with obvious fibrosis around the lesions and unclear boundary with surrounding tissues. **(B)** Lesions with fibrotic cyst wall (blue arrow) and uniform red staining lamellar internal sacs (white arrows). Case 12: **(C,D)** There are fibrosis (blue arrows) of the cyst walls with calcification and inflammation (black arrows) at the periphery.

**Figure 5:**
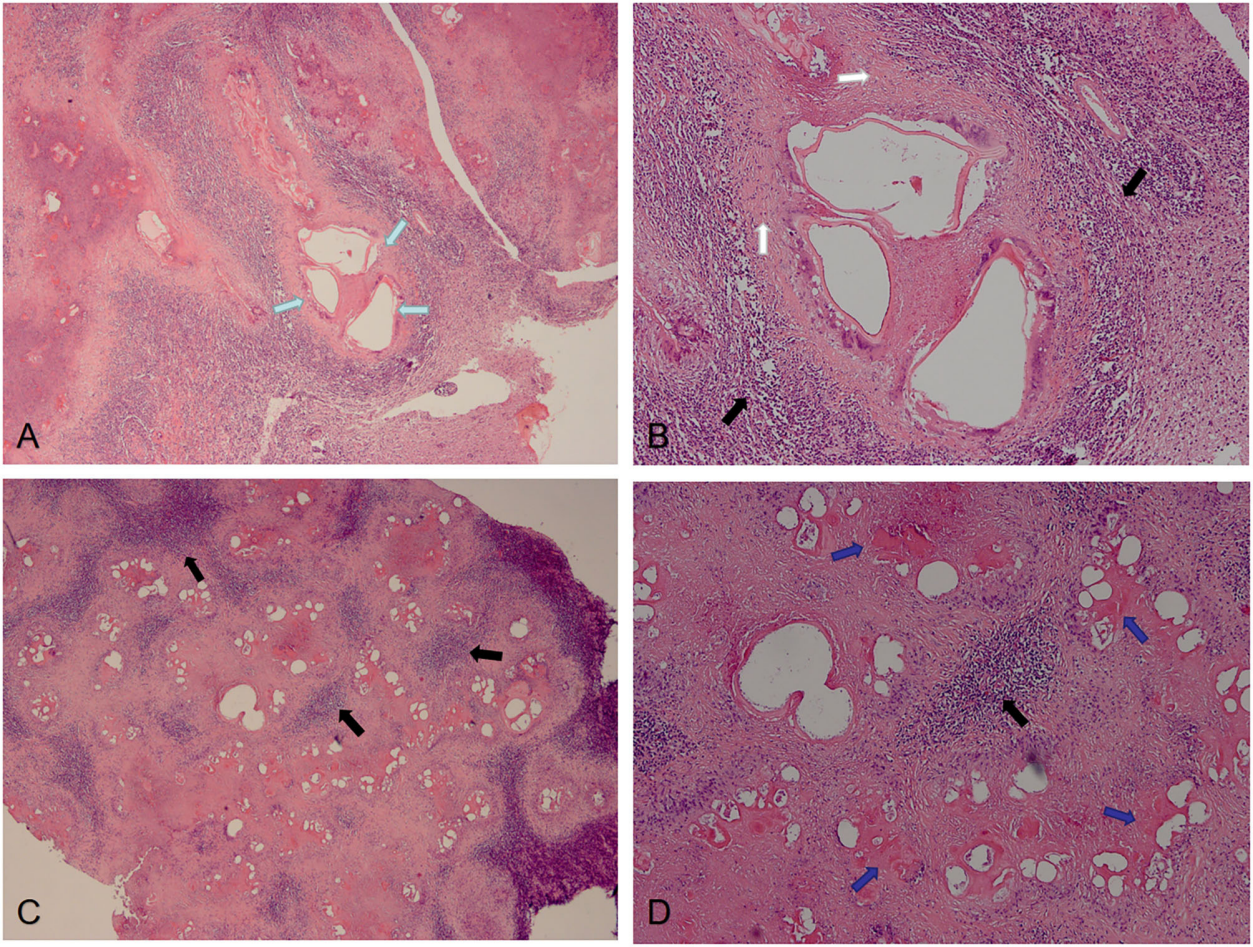
Pathological features of IAE. Case 14: **(A,B)** Multiple vesicular fusion lesions of varying sizes (green arrows). These lesions are separated from the surrounding tissue by fibrous tissue (white arrows). The area of inflammatory cell infiltration (black arrows) is formed around these lesions. Case 13: **(C,D)** Lesions are diffusely infiltrating with necrosis (blue arrows) that destroy the surrounding parenchyma.

The postoperative pathological findings of four patients with IAE are showed in Figures 4, 5. HE staining demonstrates vesicular lesions with several internal sacs; fibrosis and calcification are seen in the wall of these capsules (Figure 4). Multiple vesicular fusion lesions of varying sizes are showed in Figure 5. These lesions are diffusely infiltrating with necrosis that destroy the surrounding parenchyma. Besides, atrophy is seen in the brain parenchyma with marked inflammatory response.

### Treatment and Outcomes

We summarized the information about treatment and outcomes regarding these patients in Tables 1–3. All of the 21 patients experienced AE surgery: six underwent cerebral AE surgery (Table 1), five underwent cerebral and hepatic AE surgery (Table 2), and 10 only underwent hepatic AE surgery (Table 3). Among the 11 patients who underwent cerebral AE surgery, five of them underwent partial resection with a mortality rate of 60.0%, and six patients received total resection with a mortality rate of 33.3%. Two (33.3%) patients with total resection of cerebral AE lesions experienced postoperative recurrence. It is worth noting that one patient (case 16) with primary IAE who underwent radical surgery developed brain and liver recurrences 11 years after surgery. For medicine treatment, patients generally had poor compliance with albendazole (ABZ). Among the 21 patients, seven patients took ABZ and traditional Tibetan medicine intermittently, four patients refused to take ABZ, and only 10 patients took ABZ regularly at a dosage of 0.8 mg/d (divided in two doses). The duration of patients took albendazole ranged from 2 months to 3 years.

**Table 2:** Clinical data of patients who underwent both cerebral and hepatic alveolar echinococcosis (AE) operation.

**Case no**.	**Age/ sex**	**Main symptoms**	**Cerebral location**	**Other location**	**Treatment**	**Cerebral AE surgery**	**Degree of cerebral surgery**	**Duration of ABZ therapy (Regularity)**	**AE duration (mths)^a^**	**Max mRS/ GCS at post-IOp**	**Time to recurrence**	**Outcomes (follow-up^b^)**
8	46/M	Epilepsy	Left PO	Liver, Lung	HOp + ABZ +IOp	Left occipital and parietal lobectomy	Completely removed	3 years (Regular)	60 mths	2/15	1 mth	Alive (20 mths)
9	49/M	Headache, Dizziness	Left O	Liver, Lung, Kidney	Hop + ABZ + IOp	Left occipital lobectomy	Completely removed	3 years (Regular)	72 mths	0/15	None	Alive (22 mths) Headache
13	24/F	Headache, Epilepsy, Vomiting	Multiple	Liver, Lung	HOp + ABZ + IOp	Left frontal and occipital lobectomy	Partially removed	2 years (Regular)	108 mths	NA	NA	Dead (39 mths)
18	51/F	Headache, Dizziness	Multiple	Liver, Lung	HOp + ABZ +IOp	Left occipital and parietal lobectomy	Partially removed	1 years (Regular)	132 mths	NA	NA	Dead (21 mths)
21	69/F	Stomachache	Right thalamus	Liver	ABZ + HOp + IOp	Right thalamic lobectomy	Completely removed	1 years (Regular)	24 mths	NA	None	Dead (24 mths)

*F, female; M, male; T, temporal lobe; O, occipital lobe; PO, parieto-occipital lobe; AE, alveolar echinococcosis; IOp, intracranial AE operation; HOp, hepatic AE operation; ABZ, albendazole; Max, maximum; mRS, modifified Rankin Scale; GCS, Glasgow coma scale; mth, month; NA, not applicable*.

a *Course of disease, duration from AE onset until death or last follow-up (months)*.

b *Follow-up, duration of follow-up after discharge (months)*.

**Table 3:** Clinical data of patients who underwent hepatic alveolar echinococcosis (AE) operation.

**Case no**.	**Age/sex**	**Main symptoms**	**Cerebral location**	**Other location**	**Treatment**	**Duration of ABZ therapy (Regularity)**	**AE duration (mths)^a^**	**Max mRS/ GCS at post-HOp**	**Outcomes (follow-up^b^)**
2	28/M	Headache, Stomachache	Multiple	Liver	HOp	6 months (Irregular)	41 mths	0/15	Alive (23 mths)
4	37/F	Headache	Multiple	Liver, Lung	HOp +ABZ	3 years (Regular)	36 mths	0/15	Alive (24 mths)
5	37/M	Headache	Left P	Liver	HOp	None	36 mths	0/15	Alive (6 mths)
6	42/M	Headache, Vomiting, Epilepsy	Multiple	Liver	HOp	None	119 mths	NA	Dead (35 mths)
7	49/F	Stomachache	Right T	Liver	HOp + ABZ	2 years (Regular)	92 mths	0/15	Alive (20 mths)
10	52/M	Stomachache	Multiple	Liver	HOp+ABZ	3 years (Irregular)	36 mths	0/15	Alive (6 mths)
15	37/M	Headache	Multiple	Liver	Liver transplantation + ABZ	1 year (Regular)	23 mths	NA	Dead (14 mths)
17	43/F	Headache, Vomiting	Multiple	Liver, Lung	HOp	None	60 mths	NA	Dead (46 mths) Unknown
19	51/F	Headache, Stomachache	Multiple	Liver	HOp+ABZ	1 year (Irregular)	30 mths	NA	Dead (30 mths)
20	63/M	Headache	Multiple	Liver	HOp+ ABZ	2 years (Regular)	120 mths	NA	Dead (41 mths)

*F, female; M, male; T, temporal lobe; P, parietal lobe; AE, alveolar echinococcosis; IOp, intracranial AE operation; HOp, hepatic AE operation; ABZ, albendazole; Max, maximum; mRS, modifified Rankin Scale; GCS, Glasgow coma scale; mth, month; NA, not applicable*.

a *Course of disease, duration from AE onset until death or last follow-up (months)*.

b *Follow-up, duration of follow-up after discharge (months)*.

With a median follow-up duration of 22 months (range: 3–46 months), 10 patients died during follow-up with an overall mortality rate of 47.6%. In total, 28.9% of the patients died within 5 years after AE diagnosis, and 71.6% died within 10 years. Therefore, the cumulative 10-years survival rate was therefore 28.4% in the present series. The median interval between the date of diagnosis of AE to death was 84 (19-144) months. Of the 10 patients, four died of cerebral hernia and intracranial hypertension (cases 15, 18, 19, and 21), one died of cirrhosis of the liver and hepatic encephalopathy (case 6), two died of respiratory failure (cases 16 and 20), but the causes of death were unknown in remaining three patients (cases 13, 14, and 17). Of the 11 patients who survived at follow-up, three had moderate sequelae such as monoplegia (case 12) and decreased vision (case 1 and case 11) that mildly affected function, but they were compatible with independent living. Two patients (case 8 and case 12) reported seizures at follow-up.

## Discussion

As far as parasitic zoonoses are concerned, echinococcosis is one of the most important infectious diseases on the Tibetan Plateau in Sichuan, China (17–20). Cerebral metastases occur in only 1% of the infected AE patients (21). Our study reports a series of 21 cases of individuals diagnosed with IAE. These patients had a high mortality rate of 47.6% during follow-up for a median follow-up duration of 22 months. We summarized the clinical and radiological characteristics and outcomes among patients with IAE.

The mean age at diagnosis was 41.8 years in our study. Some studies showed that the mean age of diagnosis for IAE is 50–60 years (4, 14, 22). Other studies reported the age of diagnosis are under 40 years (12, 23). However, the patients with cerebral CE (caused by *E. granulosus*) are usually diagnosed at a younger age, and most were under the age of 20 years (6, 24–26). Farmers were at a high risk of infection in our cases, which is similar to the study that reported 65% of cases were attributable to farming (27). Forestry, hunting, or simply living in a disease cluster or rural setting are risk factors for infection (27, 28). The most common risk factor was farming or simply living in a rural setting (4). AE prevalence in the semi-nomadic group is higher than in the fully nomadic cohort (29, 30). The higher AE prevalence in farmers may result from contact with dogs or with a dog's environment. Monks or Buddhist nuns (23.8%) in the present study were also at a high risk of infection, which may be due to monks' contact with stray dogs that feed around temples (8, 9). Furthermore, we found that more than 95% of patients were illiterate, which implies that the prevalence of echinococcosis may have a relationship with the education level. Education can determine occupational choices and lifestyle to a certain extent (8).

Clinical manifestations and disease courses vary profoundly for patients. Previous studies reported that 70–80% of the patients were symptomatic at first admittance (22, 28). Headache was the most common symptom reported in previous studies as well as in ours (12). Other symptoms, such as epilepsy, visual field alteration, dysarthria, and hemiparesis, vary with the intracranial location of the disease, with non-specific clinical features (12, 23, 31). Patients with hepatic AE, especially those preparing for surgery, are routinely screened for head and chest conditions. Therefore, some patients diagnosed with IAE may not have neurological symptoms. Clinical manifestations associated with AE resemble those of a “malignant” disease, with a slow-growing pattern, local tissue infiltration, and metastases (11). There can be a clinical latency of 5 to 15 years after initial infection (12). Consequently, asymptomatic patients can already be infected with AE in the brain, but the disease may not manifest until the cerebral lesion becomes quite large.

The liver and lungs were the most commonly involved extracranial viscera in our study. Previous studies showed that almost all primary AE lesions occur in the liver (6). Primary extrahepatic disease without liver involvement is probably very rare (32, 33). In addition, brain metastases are primarily associated with hepatic lesions (33, 34). Human echinococcosis is usually caused by an intra-hepatic growth of the parasitic larvae. When an individual becomes infected, most oncospheres penetrate the mucosa and circulate in the blood, which is filtered by the liver (35). Thus, the liver is usually the site of primary disease, and metacestode material may spread to other organs (such as the lungs or brain) via lymph- or blood vessels (22, 28, 32). It is generally recommended that a radiological examination be performed to exclude pulmonary and cerebral AE in patients having hepatic AE (16).

Most of the AE lesions are situated at the level of the cerebral hemisphere, and parietal lobe is the most common location for cerebral echinococcosis (23). Consistent with other studies (12, 14, 36, 37), the typical neuroimaging features are low-signal shadow lesions in T2-weighted images with multiple small vesicles inside the lesions, and ring or cauliflower-like contrast enhancement around these lesions. Cerebral echinococcosis with multiple brain lesions may be confused with a tuberculoma, metastatic tumor disease, or brain abscess (14, 23, 33). However, these characteristic brain MRI appearances are helpful in the differential diagnoses (14, 23). A tuberculoma has nodular homogenous enhancement and metastatic tumors harbor enhanced nodules; peripheral edema around the ring enhancement is seen in abscesses, nevertheless, the abscess is hyperintense on T2-weighted MRI, whereas alveolar echinococcosis is hypointense (23).

The imaging features of hepatic AE are also important, as the primary lesions (usually hepatic AE) play a critical role in the diagnosis of IAE (13). In this study, there was usually no enhancement of the hepatic lesion of AE with contrast CT scanning, indicating lack of blood supply to the parasite. Due to this lack of blood supply, there is frequently necrosis and liquefaction in the center of the hepatic lesion, which leads to formation of pseudocystic cavities (38). In addition, the involvement of porta hepatis was common, and the portal vein was the most commonly involved vessel. Infiltration of the common bile duct and portal vein may lead to obstructive jaundice, splenomegaly, esophageal varices, portal cirrhosis, and portal hypertension (38).

Immunoblotting tests of serology are the mainstay of non-invasive diagnosis in alveolar echinococcosis. Serological test (ELISA) has yielded sensitivity over 90% and with a specificity over 95%, but it remains unclear whether the same rates apply to extrahepatic alveolar echinococcosis (16). Serological interpretation can be difficult in cases of extrahepatic manifestation and sometimes remains insufficient to differentiate AE from CE (39). Furthermore, serology was more often negative in AE patients with immunosuppression (40). Overall, 16 cases were confirmed by serological tests and 14 were positive and 2 were weekly positive in our study. Except for serological tests, cerebral MRI and pathology is needed for the diagnosis of IAE.

Treatment includes drug therapy and surgery. A considerable number of patients in the present study did not take medication regularly (intermittent use of albendazole), chose to use “traditional Tibetan medicine” for religious reasons, or even refused to take albendazole. The duration of patients took albendazole ranged from 2 months to 3 years. Besides, some patients took medications intermittently (take for 1 month, discontinue for 1 week). Reuter et al. suggested that continuous treatment is safe, and intermittent treatment should no longer be used (41). Expert consensus on the diagnosis and treatment of human AE also suggests that benzimidazole is necessary for all inoperable patients and following AE surgery; continuous ABZ treatment is well-tolerated and has been applied for more than 20 years in some patients (16). The poor drug compliance of patients may be attributed to a lack of adequate understanding of AE among patients, as well as a lack of instruction on the importance of continuous treatment. Therefore, lifelong Albendazole treatment is recommended for patients with IAE; patients' medication compliance needs to be improved through enhancing health education and regular telephone follow up. Further randomized clinical trials are needed to standardize the drug treatment of patients with IAE, test the pursued effectiveness of ABZ and developing new drugs.

Surgery in alveolar echinococcosis is associated with operative complications and the spread of disease (4). Radical surgery is the preferred treatment for alveolar echinococcosis (most of them were hepatic AE) (16, 42, 43). More specifically, the best treatment is a radical resection with a safety margin of 2 cm (4). However, it is almost impossible for patients with multifocal cerebral lesions and accompanied by extracranial viscera. In our cohort, 10 patients who received cerebral AE surgery had extracranial viscera, thus these surgeries were considered palliative. The prognosis of these patients who underwent palliative surgery did not improve significantly. However, one patient with primary IAE undergoing radical surgery had a better prognosis. It took him 11 years after the surgery to develop a recurrence of his brain and liver. Therefore, radical surgery could be considered as a curative approach in primary IAE (which is extremely rare) but should be weighed against ABZ-treatment and post-operative sequelae; palliative surgery could be considered for cerebral disease progression under ABZ treatment and symptoms that cannot be controlled by other means. In addition, due to the rarity of the disease and the complicated conditions of each patient, interdisciplinary discussion is needed to provide tailored management for each patient with AE (4), and long-term follow-up are need to monitor the possibility of recurrence (43).

The prognosis of different hydatid types varies greatly. The mortality rate is 10% for cerebral CE, the recurrence rate of surgery-only is 13%, and the recurrence rate of surgery with chemotherapy approaches is 4% (31). However, the 10-years fatality rate for AE exceeded 90% in earlier untreated cohorts (44). In some countries, the prognosis of patients with AE appears to have improved as a result of changes in treatment and management. A long-term follow-up study of 117 patients from eastern France showed that the 5-years actuarial survival rate increased from 67 to 88% (21). In 1970, life expectancy in Switzerland was estimated to be reduced by 18.2 years for men and 21.3 years for women, but it was reduced to ~3.5 and 2.6 years by 2005, respectively (45). Nevertheless, the 10-years survival rate after AE diagnosis was 28.4% in our cohort, which is similar to the 29% of untreated patients reported in the previous study (44). Despite significant advances in diagnostic and therapeutic methods, the treatment of IAE results in unsatisfactory outcomes. The high mortality rate of IAE may be associated with difficult surgical treatment in the terminal stage of AE and poor medication compliance.

Our study had several limitations. First of all, it was a hospital-based, retrospective, observational study, which is prone to selection bias due to the lack of randomization. Children younger than 14 years of age are often admitted to the Pediatric Department; thus, most of them were not included in this study. Nonetheless, this study provides useful information of adult for future studies, which can help guide the development of management strategies for human alveolar echinococcosis in the Tibetan region. Secondly, many patients demonstrated poor drug compliance, which may affect the efficacy of drug treatment and make it difficult to study the effects of ABZ. Finally, since this study only included AE patients confirmed by surgical pathology and excluded probable/possible AE cases, it may affect the outcome of mortality and survival rate. Prospective studies using standard therapy and long-term follow-up are required for the further development of preventive and therapeutic strategies for IAE.

## Conclusion

IAE is a severe infection with high mortality and postoperative recurrence rates. Further understanding the typical neuroimaging characteristics is helpful for the diagnosis of IAE. Despite substantial advances in diagnostic and therapeutic methods, the treatment of IAE remains difficult and results in unsatisfactory outcomes. The major critical issue is surgical treatment of IAE although the disease is disseminated. Besides, lifelong albendazole would be indicated, but most patients had poor medication compliance and uncertain efficacy. Patients' medication compliance needs to be improved, such as enhancing health education and regular telephone follow-up. Further studies are needed to standardize the treatment of patients with IAE.

## Data Availability Statement

The datasets generated for this study are available on request to the corresponding author.

## Ethics Statement

The studies involving human participants were reviewed and approved by Ethics Committee of Ganzi Tibetan Autonomous Prefecture People's Hospital. The patients/participants provided their written informed consent to participate in this study.

## Author Contributions

DZ designed the study. SL, JiaC, and YH carried out the study, collected data, and analyzed data. SL, JiaC, YH, YD, JieC, WF, ZZ, AA, and YL helped with data collection. BY gave advice and help in revising the article. SL analyzed the results and wrote the manuscript. JiaC and DZ revised the final version. All authors contributed to the article and approved the submitted version.

## Conflict of Interest

The authors declare that the research was conducted in the absence of any commercial or financial relationships that could be construed as a potential conflict of interest.
